# High Resolution Mass Spectroscopy-Based Secondary Metabolite Profiling of *Nymphaea nouchali* (Burm. f) Stem Attenuates Oxidative Stress via Regulation of MAPK/Nrf2/HO-1/ROS Pathway

**DOI:** 10.3390/antiox10050719

**Published:** 2021-05-03

**Authors:** Md Badrul Alam, Marufa Naznin, Syful Islam, Fanar Hamad Alshammari, Hee-Jeong Choi, Bo-Rim Song, Sunghwan Kim, Sang-Han Lee

**Affiliations:** 1 Department of Food Science and Biotechnology, Graduate School, Kyungpook National University, Daegu 41566, Korea; mbalaml@knu.ac.kr (M.B.A.); alfnar@knu.ac.kr (F.H.A.); chj1901@knu.ac.kr (H.-J.C.); sbr9707@knu.ac.kr (B.-R.S.); 2 Food and Bio-Industry Research Institute, Inner Beauty/Antiaging Center, Kyungpook National University, Daegu 41566, Korea; 3 Department of Chemistry, Kyungpook National University, Daegu 41566, Korea; naznin@knu.ac.kr (M.N.); syful@knu.ac.kr (S.I.); 4 Mass Spectroscopy Converging Research Center and Green-Nano Materials Research Center, Daegu 41566, Korea; 5 Knu BnC, Daegu 41566, Korea

**Keywords:** antioxidant, *Dillenia indica*, heme oxygenase 1 (HO-1), nuclear factor erythroid 2-related factor 2, RAW264.7 cells

## Abstract

The secondary metabolites profiling of *Nymphaea nouchali* stem (NNSE) extract was carried out using a high-resolution mass spectroscopic technique. The antioxidant effects of NNSE, as well as the underlying mechanisms, were also investigated in tert-butyl hydroperoxide (*t*-BHP)-stimulated oxidative stress in RAW264.7 cells. Tandem mass spectroscopy with (−) negative mode tentatively revealed the presence of 54 secondary metabolites in NNSE. Among them, phenolic acids and flavonoids were predominant. Phenolic acids (brevifolincarboxylic acid, *p*-coumaroyltartaric acid, niazinin B, lalioside, 3-feruloylquinic acid, and gallic acid-*O*-rutinoside), flavonoids (elephantorrhizol, apigenin-6-C-galactoside 8-C-arabinoside, and vicenin-2), sialic acid (2-deoxy-2,3-dehydro-N-acetylneuraminic acid), and terpenoid (α-γ-onoceradienedione) were identified in NNSE for the first time. Unbridled reactive oxygen species/nitrogen species (ROS/RNS) and redox imbalances participate in the induction and development of many oxidative stress-linked diseases. The NNSE exhibited significant free radical scavenging capabilities and was also able to reduce *t*-BHP-induced cellular generation in RAW264.7 cells. The NNSE prevented oxidative stress by inducing the endogenous antioxidant system and the levels of heme oxygenase-1 (HO-1) by upregulating Nrf2 through the modulation of mitogen-activated protein kinases (MAPK), such as phosphorylated p38 and c-Jun N terminal kinase. Collectively, these results indicate that the NNSE exhibits potent effects in preventing oxidative stress-stimulated diseases and disorders through the modulation of the MAPK/Nrf2/HO-1 signaling pathway. Our findings provide new insights into the cytoprotective effects and mechanisms of *Nymphaea nouchali* stem extract against oxidative stress, which may be a useful remedy for oxidative stress-induced disorders.

## 1. Introduction

Reactive oxygen and nitrogen species (ROS/RNS) are important for maintaining cellular homeostasis, but unbridled ROS/RNS and redox imbalances participate in the induction and development of many oxidative stress-linked diseases, including cancer, inflammation, and cardiovascular disease [1]. Tert-butyl hydroperoxide (t-BHP) is a lipid hydroperoxide analog that acts as a pro-oxidant to cause lipid peroxidation of cell membrane phospholipids resulting in changes in membrane structure and function. Thus, this model is widely accepted to assess the mechanisms responsible for oxidative stress in cells and tissues [2]. Although macrophages protect cells from various infectious agents, upon stimulation, they can generate ROS and RNS and thereby trigger epigenetic alterations, leading to the pathogenesis of chronic diseases [3]. Thus, activated macrophage models are widely accepted for the identification of active compounds and the development of functional diets through a multi-targeted strategy. Phytochemicals with intrinsic antioxidant activity can directly or indirectly orchestrate numerous cellular protectives signaling cascades and may be used as remedies for oxidative stress-induced disorders [4]. For this reason, understanding and validating the activities of natural compounds and identifying the underlying molecular mechanisms are essential to establish their potential clinical value.

Various detoxication and antioxidant enzymes, such as heme oxygenase-1 (HO-1) and NAD(P)H quinone oxidoreductase 1 (NQO1), are dependent on the activation of nuclear factor erythroid 2-related factor 2 (Nrf2) [5]. In the resting state, cytosolic Kelch-like ECH-associated protein 1 (Keap1) causes the degradation of Nrf2 through the ubiquitin-proteasome system. Oxidative stress or xenobiotic challenge can prevent Nrf2 degradation by modifying the reactive cysteine residue of Keap1, which enables translocation of Nrf2 to the nucleus and binding to antioxidant-related elements (AREs) in the promoter regions of antioxidant and cytoprotective genes [5]. Furthermore, Nrf2 nuclear translocation is also dependent on the activation of mitogen-activated protein kinase (MAPK), phosphatidylinositol 3-kinase/Akt (PI3K/AKT), and protein kinase C (PKC) [6].

*Nymphaea nouchali,* which is commonly known as a water lily in English, and a shapla in Bangla, belongs to the Nymphaeaceae family and is regarded as the national flower of Bangladesh. Traditionally, the whole plant is used to treat liver disorders. Leaves, roots, and flowers are used as cardiotonic, astringent, demulcent, and as a remedy for kidney problems [4,7,8]. The seeds, however, are considered to be stomachic and restorative, and they are prescribed in a diet for diabetes mellitus in the Ayurvedic system of medicine [7]. A novel Ca^2+^-dependent lectin has been identified from *N. nouchali* tuber and exhibits potential antiproliferative activity [9]. In a previous study, we demonstrated that the abundance of polyphenolic compounds in *N. nouchali* flowers triggers the DNA protecting activity from *t*-BHP induced oxidative stress through the regulation of antioxidant signaling cascades [4,10]. Interestingly, the *N. nouchali* stem has not been subjected to any systematic scientific investigation to assess its antioxidant activity.

Therefore, the aim of the present study was to determine the secondary metabolite profile of the ethanolic extract of *N. nouchali* stem (NNSE) using high-resolution mass spectroscopic analysis and the antioxidant capacity in vitro. Moreover, we investigated the protective role of NNSE against t-BHP-induced oxidative stress in RAW 264.7 cells and the underlying mechanisms.

## 2. Materials and Methods

### 2.1. Plant Materials and Extraction

*N. nouchali* stem (NNS) plants were collected from Khulna in Bangladesh, identified by the National Herbarium of Bangladesh, and stored in our laboratory for future reference. To prepare an ethanolic extract, 50 g of coarse NNS powder was extracted with 500 mL of ethanol under reflux for 1 h (three times). The mixture was filtered, dried in a rotary vacuum evaporator, lyophilized, and stored at −20 °C. The ethanolic extract residue (NNSE) was dissolved in deionized H_2_O to obtain a 30 mg/mL stock solution. For ESI-MS/MS analysis, a stock solution (10 mg/mL) was prepared in 100% HPLC-grade ethanol and then diluted using a 70% ethanolic solution. Homogenization was carried out by vortexing for 1 min, followed by sonication for 5 min in a sonication bath (Powersonic 410, Hwashin Technology Co., Seoul, Korea) [11].

### 2.2. Electro Spray Ionization (ESI)-Mass Spectroscopy Analysis

A Q-Exactive Orbitrap mass spectrometer (Thermo Fisher Scientific Inc., San Jose, CA, USA) was used for negative-mode ESI-MS experiments. A 500 µL graduated syringe (Hamilton Company Inc., Reno, NV, USA) and a syringe pump (Model 11, Harvard, Holliston, MA, USA) were used to immerse the sample through the ESI source at 15 µL/min. The characteristic negative-mode ESI-MS conditions were a mass resolution of 140,000 (full width at half maximum, FWHM), sheath gas flow rate of 5, sweep gas flow rate of 0, auxiliary gas flow rate of 0, spray voltage of 4.20 kV, capillary temperature of 320 °C, S-lens Rf level, and automatic gain control of 5 E 6. Nitrogen gas with high purity (99.99%) was used for the sheath, auxiliary, and sweep gas flow. For negative modes, external calibrations were performed using a Pierce Velos solution (Thermo Fisher Scientific) in the ESI source.

Three different stepped normalized collision energies (NCE = 10, 30, and 40) were used to perform MS/MS experiments with the same instrument. The instrument was operated in (−) mode, and the other operative parameters for MS/MS experiments were as follows: sheath gas flow rate of 10, auxiliary gas flow rate of 0 (arbitrary units), spray voltage of 3.50 kV, capillary temperature of 320 °C, and an S-lens Rf level of 50 [2,12].

### 2.3. Data Processing

Mass spectrum data acquired from the orbitrap mass spectrometer were organized using Xcalibur 3.1 along with foundation 3.1 (Thermo Fisher Scientific Inc. Rockford, IL, USA). The *m/z* peaks were tentatively identified by matching their exact (theoretical) masses of deprotonated (M-H) adducts with measured *m/z* values and ESI-MS/MS fragmentation patterns from an in-house MS/MS database, and online databases such as FooDB (https://foodb.ca/, accessed date 2 April 2021) and METLIN database (https://metlin.scripps.edu/landing_page.php?pgcontent=mainPage, accessed date 2 April 2021). Compound structures were drawn using ChemDraw Professional 15.0 (PerkinElmer, Waltham, MA, USA).

### 2.4. Radical Scavenging Activity Assays

To assess the free radical scavenging capability of NNSE, DPPH, ABTS, superoxide, and hydroxyl-radical, scavenging assays were carried out using a previously described protocol [4]. Ascorbic acid, quercetin, and gallic acid were treated as standard antioxidants for DPPH, ABTS, and superoxide- and hydroxyl-radical scavenging assays, respectively. The percent inhibition was computed using the following equation:

(1) $$\mathrm{Radical}-\mathrm{scavenging}\;\mathrm{activity}\;(\%\;\mathrm{inhibition})=\left[\frac{\left({\mathrm{Abs}}_{\mathrm{control}}-{\mathrm{Abs}}_{\mathrm{sample}}\right)}{{\mathrm{Abs}}_{\mathrm{control}}}\right]\;\times \;100$$

where Abs_control_ is the absorbance of the control sample and Abs_sample_ is the absorbance of the experimental sample. All samples were analyzed in triplicate.

According to the method described by Alam et al. [2], the cupric-reducing antioxidant capacity (CUPRAC) and ferric reducing antioxidant power (FRAP) assays were used to define the reducing power capacity and were expressed as an ascorbic acid-equivalent antioxidant value (µM), using an ascorbic acid standard curve.

### 2.5. Cell Culture and Intracellular ROS Generation Assay

RAW 264.7 cells (American Type Culture Collection, Rockville, MD, USA) were cultured in Dulbecco’s Modified Eagle’s Medium supplemented with 10 % FBS and streptomycin-penicillin (100 µg/mL each; Hyclone) at 37 °C and 5% CO_2_. The cells (5 × 10^5^ cells/mL) were seeded into 96-well plates for 12 h, followed by treatment with NNSE (1–30 µg/mL) for 24 h with or without *t*-butyl hydroperoxide (t-BHP). An MTT assay and 2′,7′-dichlorofluorescein diacetate (DCFH-DA) method was used to evaluate the cellular toxicity and generation of *t*-BHP-induced ROS as a cellular oxidative stress biomarker, respectively, as described previously [2].

### 2.6. Western Blot Analysis

A radioimmunoprecipitation assay buffer was used to lyse and harvest the cells. A nuclear and cytoplasmic extraction kit (Sigma-Aldrich Co. St. Louis, MO, USA) was used to extract the nuclear and cytosolic protein. Protein content was quantified using the bicinchoninic acid protein assay kit (Pierce, Rockford, IL, USA). Equivalent amounts (30 μg) of protein were subjected to western blot analysis as described in our previous report using various antibodies (Supplementary Data, Table S1) [2].

### 2.7. Statistical Analysis

Data were expressed as the mean ± standard deviation (SD; *n* = 3). One-way analysis of variance, followed by Tukey’s multiple-comparisons test, was performed using SigmaPlot software (SigmaPlot, Ver 12.5, Systat Software, Inc., Chicago, IL, USA) to determine the significance for the differentiation and fusion indices. The hierarchy of activity (a < b < c) indicates statistical differences between the means. *p* < 0.05 was considered statistically significant.

## 3. Results and Discussion

### 3.1. Secondary Metabolite Profiling of NNSE

Secondary metabolites of NNSE were identified and characterized by ESI-MS/MS in negative mode, which is a standard procedure for revealing the structure of compounds from extracts. As shown in Table 1, there were 54 compounds identified on the basis of their MS^2^ information provided by the precursor ion’s mass, their fragments, known fragmentation patterns for the given classes of compounds, neutral mass loss, comparison with the available literature, and searching in online databases. The identified compounds were classified into phenolic acids, flavonoids, amino acids, dicarboxylic acids, fatty acids, sugar, flavoring agents, sialic acid, terpenoid, and others.

#### 3.1.1. Phenolic Acids

**Figure 1:**
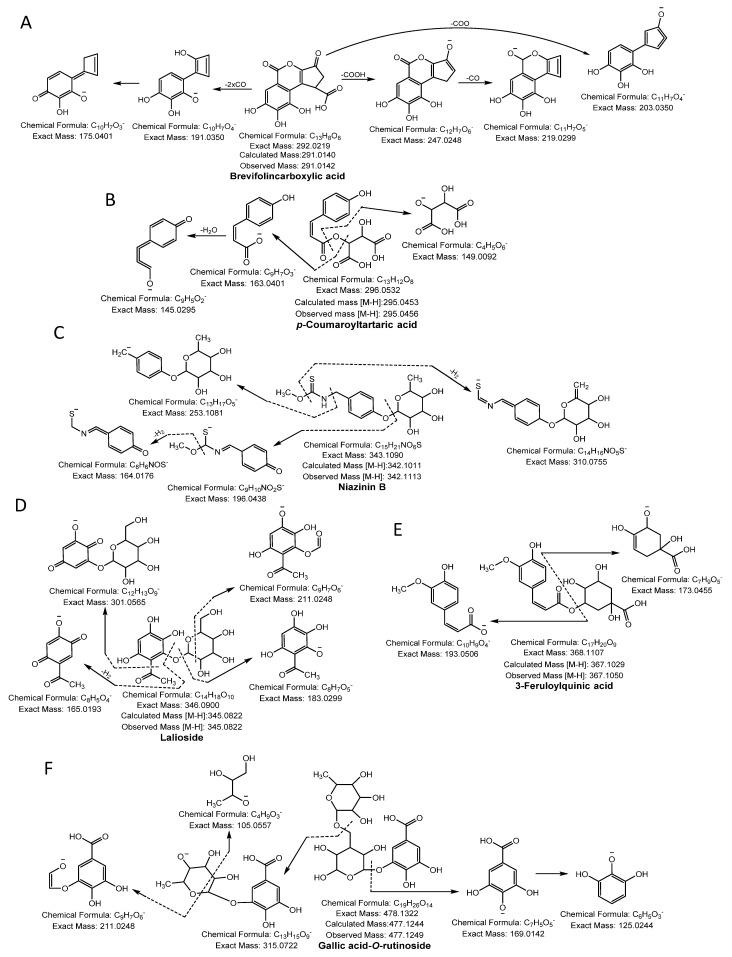
Phenolic acids (**A**) brevifolincarboxylic acid, (**B**) *p*-coumaroyltartaric acid, (**C**) niazinin B, (**D**) lalioside, (**E**) 3-feruloylquinic acid and (**F**) gallic acid-*O*-rutinoside were identified in NNSE by ESI-MS/MS.

Phenolic acid glycoside began its fragmentation by segmentation of the glycosidic bond and provided the *m/z* of the phenolic acid and the corresponding loss of sugar molecule mass (−162 Da). Moreover, phenolic acid produced its characteristic product ion by losing the neutral mass of hydroxyl (−18 Da), methyl (−15 Da), or carboxylic (−44 Da) moiety [13]. Compound 1, 2, 3, 4, and 5 yielded a molecular ion peak [M-H]^−^ at *m/z* 135.0444, 137.0227, 153.0186, 167.0344, and 169.0134 and fragmentation ions at *m/z* 91.05, 93.03, 106.02, 123.01, and 125.02, respectively, because of neutral loss of CO_2_ confirmed the presence of methyl benzoic acid, salicylic acid, protocatechuic acid, vanillic acid, and gallic acid, respectively [12,13,14]. Compound 6 produced a precursor ion peak [M-H]^−^ at *m/z* 183.0290 and diagnostic ions at *m/z* 139.0401 and 123.0088 by losing a CO_2_ and CH_3_ group and was identified as methoxygallate [15]. Compound 7 was confirmed as brevifolincarboxylic acid having a molecular ion [M-H]^−^ at *m/z* 291.0141 and yielding a daughter ion at *m/z* 247.02 by loss of CO_2_ and *m/z* 219, and 191.04 from successive losses of CO (Figure 1A). Compound 10 yielded an ion [M-H]^−^ at *m/z* 305.0300, generating a characteristic peak at *m/z* 273.01 from a loss of CH_3_OH, whereas other quasi-molecular ions were formed from a consecutive loss of CO and was identified as methyl brevifolincarboxylic acid [13,16]. Compound 8 produced a [M-H]^−^ at *m/z* 293.0300, yielded characteristic ion peaks at *m/z* 169.01 by the loss of gallic acid, and was confirmed as pyrogallol gallate [17]. Compound 9 ([M-H]^−^ at *m/z* 295.0456) was identified as *p*-coumaroyltartaric acid, showing daughter ions at *m/z* 163.04 and 149.00, which represented the loss of the tartrate and coumaroyl moiety, respectively [18] (Figure 1B). Compound 11 produced a monoisotopic mass [M-H]^−^ at *m/z* 331.0661 and yielded a daughter ion peak at *m/z* 169.0142 from a loss of glucose (−162 Da) and was identified as galloylglucose. Compound 19 had a parent ion peak at *m/z* 483.0779 [M-H]^−^ with fragment ions at *m/z* 331.0671, corresponding to loss of the galloyl moiety (−152 Da) and was identified as digalloylglucose [13]. Compound 12 was identified as niazinin B as it yielded a parent ion peak at *m/z* 342.1115 [M-H]-, generating characteristic ion peaks at *m/z* 310.07 and 196.04 resulting from the loss of MeOH and the rhamnosyl moiety (−146 Da), respectively [19] (Figure 1C). Compound 13 produced a parent ion peak [M-H]^−^ at *m/z* 345.0822, yielding characteristic fragment ion peaks at *m/z* 301.05 and 183.02 resulting from the loss of CH_3_CHO (−44 Da) and a glucose moiety (−162 Da), respectively, and was confirmed as lalioside [20] (Figure 1D). Compound 14 had a precursor ion peak [M-H]^−^ at *m/z* 353.0865, generating daughter ion peaks at *m/z* 309.09 (loss of CO_2_), *m/z* 191.05 (loss of caffeic moiety), *m/z* 179.03 (loss of quinic moiety), and *m/z* 173.04 (dehydrated quinic moiety) and was characterized as 5-*O*-caffeoylquinic acid [21]. Compound 17 exhibited an ion peak [M-H]^−^ at *m/z* 381.1204, yielding a characteristic peak at *m/z* 201.07 (loss of caffeoyl moiety), and other fragmented ions that were similar to the quasi-molecular ion of compound 14. Thus, compound 17 was considered ethyl 5-*O*-caffeoylquinic acid. Compound 15 had a quasi-molecular ion [M-H]^−^ at *m/z* 367.1050, yielding fragment ions at *m/z* 193.0506 (feruloyl moiety) and 173.0455 (dehydrated quinic moiety), and was designated 3-feruloylquinic acid [22] (Figure 1E). Compound 16 was confirmed as isoferulic acid 3-*O*-glucuronide because it generated a molecular ion peak [M-H]^−^ at *m/z* 369.0822, yielding a fragment ion at *m/z* 193.05 from loss of a glucuronide moiety (−176 Da), which was similar to the quasi-molecular ion of compound 15. Thus, compound 16 was identified as isoferulic acid 3-*O*-glucuronide [17]. Compound 18 generated a monoisotopic ion peak [M-H]^−^ at *m/z* 477.1249 and fragment ions at *m/z* 315.0722 by losing a hexose moiety (−162 Da) and at *m/z* 169.0142 (gallic acid). The glucose moiety of the daughter ion, 315.0722, was cleaved further to produce an MS^2^ spectrum at *m/z* 105.0557 and 211.0248, suggesting the compound was gallic acid-*O*-rutinoside (Figure 1F).

#### 3.1.2. Flavonoids

**Figure 2:**
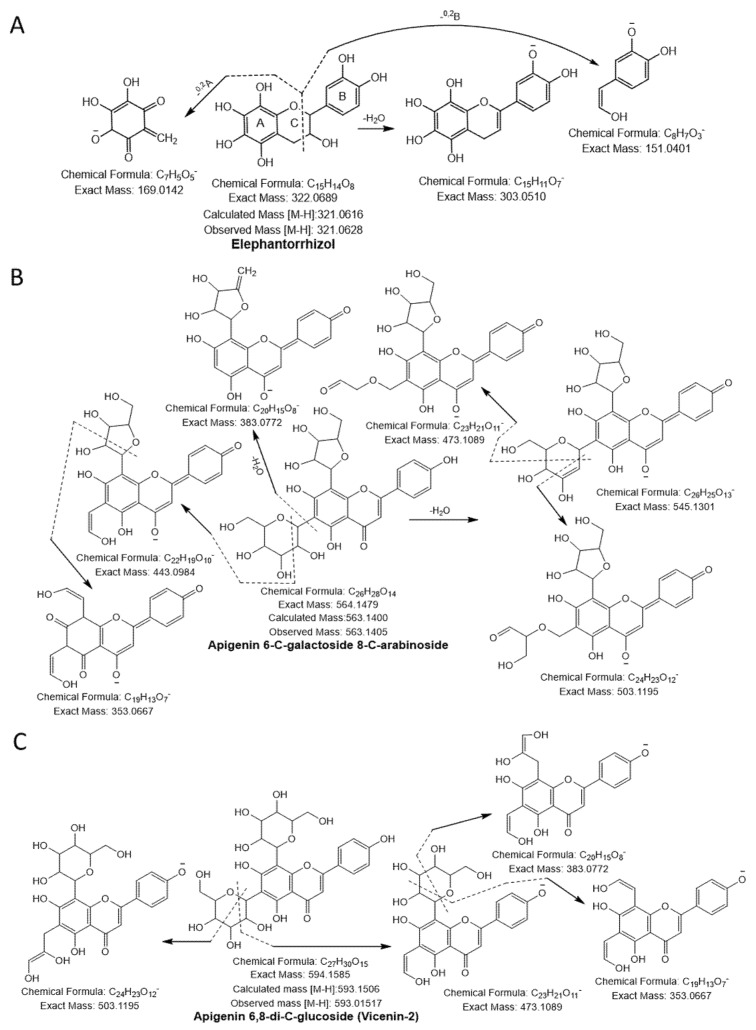
Flavonoids (**A**) elephantorrhizol, (**B**) apigenin 6-C-galactoside 8-C-arabinoside, (**C**) apigenin 6,8-di-C-glucoside (vicenin-2) were identified in NNSE by ESI-MS/M.

Compound 20 had a molecular ion [M-H]^−^ at *m/z* 289.0692, yielding a fragment ion at *m/z* 245.04 from a loss of C_2_H_4_O (−44 Da) and at *m/z* 151.04 and *m/z* 137.02 by cleavage of the C ring at the 1,3 position. The compound was confirmed as catechin [23]. Compound 21 was identified as taxifolin having a parent ion peak [M-H]^−^ at *m/z* 303.0470 with characteristic fragment ions of *m/z* 285.04 ([M-H-H_2_O]^−^), *m/z* 241.05 ([M-H-H_2_O-CO_2_]^−^), *m/z* 177.01 ([^1,4^B-2H]^−^), and *m/z* 125.02 ([^1,4^A]^−^) [24]. Compound 22 yielded a precursor ion peak [M-H]^−^ at *m/z* 321.0628 and a fragment ion at *m/z* 303.05 from a loss of H_2_O and at *m/z* 169.01 and 151.04 by cleavage of the C ring at the 1,3 position. The compound was confirmed as elephantorrhizol (Figure 2A). Compound 23 was confirmed as naringenin 7-sulfate based on the observed parental ion peak [M-H]^−^ at *m/z* 351.0175 and further verified by the MS/MS experiment, which showed a characteristic ion at *m/z* 271.06 from loss of SO_3_ (−80 Da) [24]. The monoisotopic mass [M-H]^−^ at *m/z* 563.1045 and the other characteristic peaks were evident at *m/z* 473.1089 and 503.1195 from cleavage of the pentose sugar moiety at the 0,2 and 1,3 position, respectively, and *m/z* 443.0984 (cleavage of glucose moiety 0,2 position), indicating the existence of cross hexosyl units. In addition to the peaks at *m/z* 383.0772 from the loss of the glucosyl moiety with neutral loss of H_2_O, *m/z* 353.0667 was produced from daughter ion, *m/z* 443.0984, through cleavage of a pentose sugar moiety at the 0,2 position. The predicted structure was aglycones of apigenin (Figure 2B). Hence, compound 24 was confirmed as apigenin 6-C-galactoside 8-C-arabinoside [25]. Compound 25 was designated apigenin 6,8-di-C-glucoside (Vicenin-2) because it yielded a similar quasi-molecular ion as compound 24 (Figure 2C). Based on the precursor ion peak of compound 26 ([M-H]^−^ at *m/z* 609.1460), quercetin 3-*O*-neohesperidoside was suggested because of the daughter ion peaks appearing at *m/z* 301.0354, 271.0247, and 178.9978 [26].

#### 3.1.3. Sialic Acid

**Figure 3:**
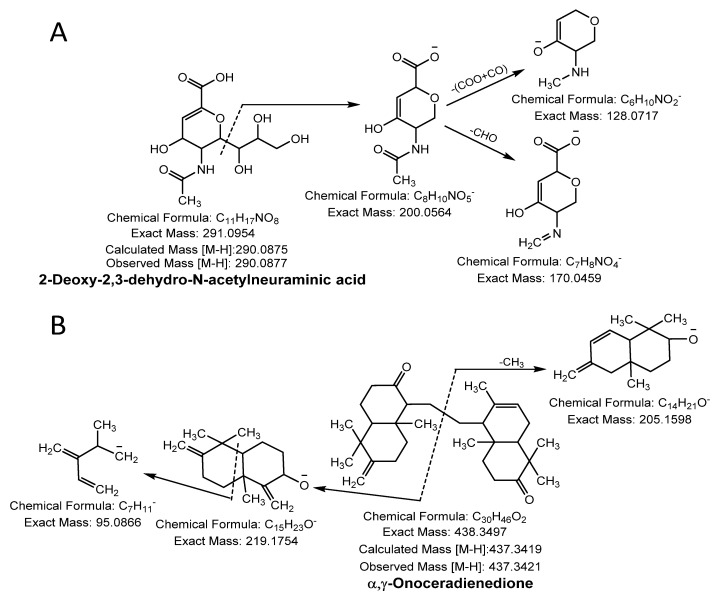
Sialic acid (**A**) and terpenoid (**B**) were identified in NNSE by ESI-MS/MS.

A molecular ion peak [M-H]^−^ at *m/z* 290.0877 (molecular formula C_11_H_17_NO_8_) and a characteristic base peak at *m/z* 200.0564 ([M-H-C_3_H_7_O_3_]^−^), further produced the MS^2^ daughter ion peak at 170.0459 and 128.0342 from a loss of -CHO (−29 Da) and C_2_O_3_ (-COO + CO; −72 Da). Thus, compound 27 was considered 2-deoxy-2,3-dehydro- N-acetylneuraminic acid (Figure 3A).

#### 3.1.4. Terpenoid

Compound 28 was identified as terpenoid with the parent ion peak [M-H]^−^ at *m/z* 437.3421 (molecular formula C_30_H_46_O_2_) and yielding fragment ions at *m/z* 219.1754 and 205.1598 from cleavage of the α and γ bond. It was confirmed as α, γ-onoceradienedione (Figure 3B).

#### 3.1.5. Dicarboxylic Acids

Compound 29, 30, and 32, with a molecular ion peak [M-H]^−^ at *m/z* 117.0175, 133.0124, and 1610450 and fragmentation ions at *m/z* 99.00, 115.00, and 143.03 from loss of H_2_O and *m/z* 73.03, 89.02, and 99.05 from a neutral loss of CO_2_ confirmed the presence of succinic acid, malic acid, and hydroxyadipic acid, respectively [12]. Compound 31 had a precursor ion [M-H]^−^ at *m/z* 147.0292, generating characteristic ions at *m/z* 133.01 ([M-H-CH_3_]^−^ and further produced the MS^2^ daughter ion peak at 115.00 and 87.00 from loss of H_2_O and COOH, respectively. The compound was identified as citramalic acid [27].

#### 3.1.6. Others

Compounds 33, 35, 36, and 37 with quasi-molecular ions [M-H]^−^ at *m/z* 129.0916, 143.1061, 195.1384, and 199.1697, were identified as fatty acids including methylcaproic acid, caprylic acid, dodecadienoic acid, and dodecanoic acid, respectively, by comparison with the literature [2,11,12].

Compound 34 and 38, with parent ion peaks [M-H]^−^ at *m/z* 131.0696, and 118.0281, yielded daughter ion peaks at *m/z* 87.08 and 74.02 from loss of CO_2_ (−44 Da) and *m/z* at 113.06 and 100.05 from a loss of NH_2_ (−16 Da) and H_2_O (−18 Da). They were identified as ethyl-β-hydroxybutyric acid and α-amino-β-hydroxybutyric acid, respectively [28]. Compound 39 was designated pyroglutamic acid as it generated a monoisotopic peak [M-H]^−^ at *m/z* 128.0336 and a characteristic peak at *m/z* at 82.0298 from a loss of 46 Da ([M-H-2H-COO]) [12].

Compound 40, 41, and 42 with monoisotopic mass ions [M-H]^−^ at *m/z* 125.0227, 139.0213, and 231.1748 were identified as the flavoring agents, maltol, kahweofuran, and cetone V, respectively, based on the literature [2,11,12] and compared with data from the METLIN database (https://metlin.scripps.edu/landing_page.php?pgcontent=mainPage, accessed date 2 April 2021) and FooDB (https://foodb.ca/, accessed date 2 April 2021). Compounds 43, 44, and 45 yielded precursor ion peaks [M-H]^−^ at *m/z* 149.0445, 179.0553, and 341.1087 and were confirmed as the sugar compounds L-arabinofuranose, glucose, and 6-*O*-β-D-galactopyranosyl-D-galactose, respectively [2,12]. Based on the literature [2,11,12] and compared with data from the FooDB (https://foodb.ca/, accessed date 2 April 2021) and METLIN database (https://metlin.scripps.edu/landing_page.php?pgcontent=mainPage, accessed date 2 April 2021), the precursor ion peaks [M-H]^−^ at *m/z* 105.0184, 111.0443, 121.0278, 135.0444, 138.0189, 165.0395, 173.0445, 191.0553, and 242.1756, corresponding to compound 46, 47, 48, 49,50, 51, 52, 53, and 54 were identified as glyceric acid, sorbic acid, salicylaldehyde, methyl benzoic acid, hydroxynicotinic acid, ribonic acid, shikimic acid, quinic acid, and N-undecanoylglycine.

### 3.2. Radical Scavenging Activities of NNSE Extracts

**Figure 4:**
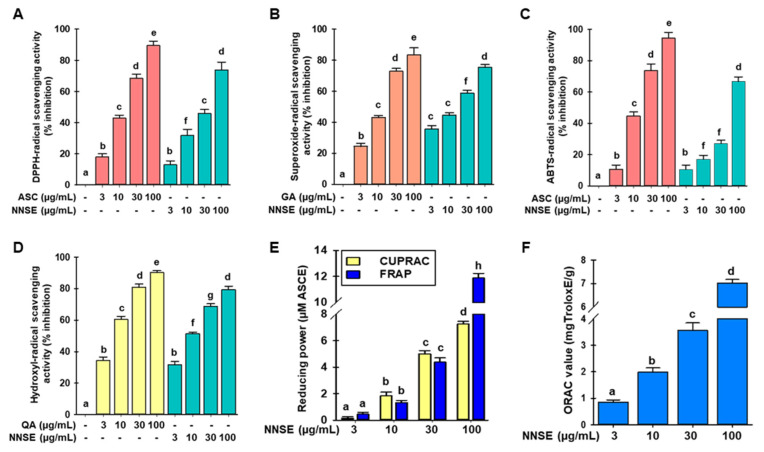
Effects of *N. nouchali stem* extract (NNSE) on radical scavenging. (**A**) DPPH-; (**B**) superoxide-; (**C**) ABTS-, and (**D**) hydroxyl-radical scavenging potential of NNSE. Ascorbic acid, quercetin, and gallic acid were designated as positive controls. (**E**) CUPRAC and FRAP; (**F**) ORAC assays were performed to assess the reducing power of NNSE. The ascorbic acid equivalent (µM) reducing power was computed for the CUPRAC and FRAP assays, whereas the ORAC potential is presented as mg Trolox equivalents/g. Values are expressed as the mean ± SD (*n* = 3), and different letters are considered statistically significant (*p* < 0.05) with one another. ASC, ascorbic acid; GA, gallic acid; QA, quercetin; ASCE, ascorbic acid equivalent. The different color represents the positive control and samples in each experiment.

The antioxidant activities of phytochemicals involve various molecular mechanisms. Thus, various methods should be used to assess the antioxidant potential of plant extracts. In this study, the antioxidant potential of NNSE was analyzed using DPPH-, ABTS-, superoxide- and hydroxyl-radical scavenging assays, along with FRAP, CUPRAC, and ORAC assays. As shown in Figure 4A,B, NNSE exhibited a dose-dependent and significant DPPH- and superoxide-radical scavenging potential with IC_50_ values of 44.59 ± 1.29 µg/mL and 18.50 ± 0.40 µg/mL, whereas the positive control, ascorbic acid, had an IC_50_ value of 16.58± 0.24 µg/mL in the DPPH-radical scavenging assay and gallic acid had an IC_50_ value of 14.34 ± 0.70 µg/mL in the superoxide-radical scavenging assay, respectively. These results suggest that NNSE exhibits antioxidant potential through a hydrogen atom transfer mechanism. Moreover, Figure 4C,D show that NNSE has a significant and concentration-dependent ability to scavenge ABTS- and hydroxyl-radical with IC_50_ values of 73.51 ± 1.07 µg/mL and 9.48 ± 0.36 µg/mL, respectively, whereas ascorbic acid and quercetin (positive control) had an IC_50_ value of 14.49 ± 0.55 µg/mL for the ABTS-radical scavenging assay and 4.13 ± 0.06 µg/mL for the hydroxyl-radical scavenging assay. Based on these results, we speculated that NNSE also uses a single electron transfer mechanism to perform its antioxidant activity. Furthermore, CUPRAC, FRAP, and ORAC assays were performed to assess the reducing capability of NNSE. NNSE exhibited 7.42 ± 0.10 and 11.69 ± 0.26 µM ascorbic acid equivalents reducing power in the CUPRAC and FRAP assays at 100 µg/mL, respectively (Figure 4E). NNSE exhibited 7.02 ± 0.56 mg Trolox equivalents/g antioxidant potential at 100 µg/mL in the ORAC assay (Figure 4F).

In recent decades, polyphenolic-rich natural nutritional regimens with antioxidant activity have nurtured concern in nutrition and food science. Among the various plant secondary metabolites, natural phenolic and flavonoid compounds are important, judging from the virtue of their antioxidant activities by free radical inhibition, peroxide decomposition, metal inactivation, or oxygen scavenging in biological systems, and they ameliorate the effects of oxidative diseases. The total phenolic and flavonoid content of NNSE was 128.11 ± 2.38 mgGAE/g (mg gallic acid equivalent per gram dry extract) and 14.37 ± 1.76 mgCAE/g (mg catechin equivalent per gram dry extract), respectively, as derived from a calibration curve (Y = 0.0541X + 0.0008, R^2^ = 0.994) of gallic acid (0–50 µg/mL) and (Y = 0.0121X + 0.0031, R^2^ = 0.993) catechin (0–50 µg/mL) (supplementary data, Figure S1). Our previous studies revealed a significant amount of total phenol in *Nymphaea nouchali* flowers and leaves at 413.02 ± 3.01 and 258.09 ± 1.31 mg GAE/g, respectively [4,29]. Furthermore, other studies have revealed that *N. alba*, *N. caerulea* flower, *N. lotus* flower, *N. pubescens* flower, and *N. stellate* flower are rich in polyphenolic compounds and show strong antioxidant activities [30,31,32,33]. Based on the literature, phenolic acids such as salicylic acid, protocatechuic acid, vanillic acid, gallic acid, brevifolincarboxylic acid, methyl brevifolincarboxylic acid, galloylglucose, lalioside, and 5-O-caffeoylquinic acid have the potential to exhibit radical scavenging effects with IC_50_ values of 10.78 ± 1.23, 10.94 ± 0.51, 4.92 ± 0.27, 7.59 ± 1.25, 4.62 ± 0.65, 3.00 ± 0.15, 6.00 ± 0.24, 7.14 ± 1.02, 10.00 ± 0.57, and 13.8 ± 1.08, respectively. In addition, among the flavonoids, catechin exhibited the highest DPPH-radical scavenging activity with an IC_50_ value of 6.38 ± 0.85, whereas the others showed activity in the following order: taxifolin > elephantorrhizol > vicenin-2 > apigenin-6-C-glactoside-8-C-arabinoside [34,35,36,37,38,39].

Traditionally, in Far East Asian countries (Korea, China, Japan, etc.), water lilies and lotus plants, including the leaves, stems, seeds, etc., have represented important food resources at temples since ancient times (Lotus flowers or the leaves are known as a plant that often appears in Buddhist events and related books). Various polyphenolic compounds in plants today have a strong potential to enhance antioxidant, antibacterial, and anti-inflammatory activities. Each part appears to play a key role in maintaining food safety, providing protection from contaminating bacteria and other pathogens [4,10]. Rice or main dishes are sometimes served on leaves instead of bowls or plates because they anticipated these biological activities experientially by packaging the daily food.

### 3.3. Attenuation of t-BHP Induced Cellular Oxidative Stress by NNSE

**Figure 5:**
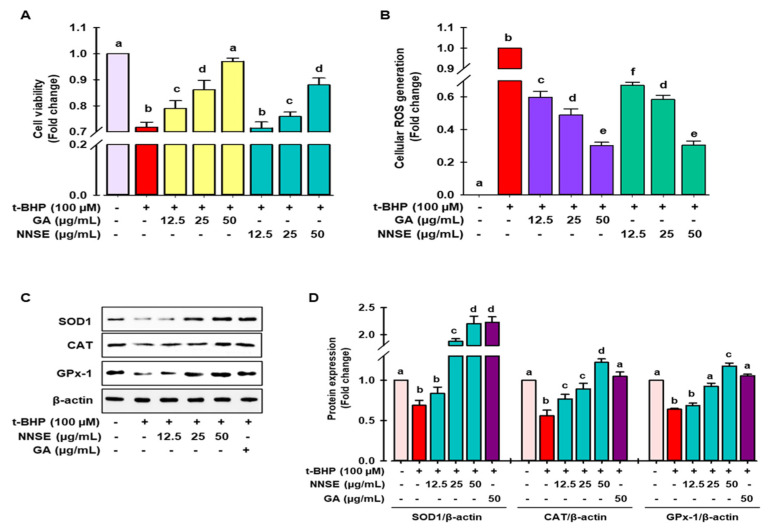
Attenuation of *t*-BHP-induced cell toxicity and intracellular ROS generation by NNSE and upregulation of antioxidant enzymes. Pretreatment of cells with NNSE and gallic acid for 12 h followed by treatment with 100 μM t-BHP for 6 h. The viable cells were counted, and the generation of cellular ROS was evaluated by (**A**) MTT assay and (**B**) DCFH-DA method, respectively. (**C**) Protein expression of SOD1, catalase, and GPx-1 was measured by western blot analysis. (**D**) The relative protein expression was quantified by Image J software. Values are expressed as the mean ± SD (*n* = 3), and different letters are considered statistically significant (*p* < 0.05) to one another. GA, gallic acid. The different color represents the model control, positive control, and samples in each experiment.

Cellular oxidative stress was induced by *t*-BHP, a short-chain lipid peroxide analog, and is widely accepted as a model to evaluate the alteration of cellular mechanisms caused by oxidative stress in cells and tissues [40]. As shown in Figure 5A, treatment with *t*-BHP caused significant cell death, whereas pretreatment with NNSE and gallic acid attenuated the cellular toxicity at nontoxic doses (supplementary data S2). Furthermore, Figure 5B shows that NNSE exhibits the capability of mitigating the production of cellular ROS in a dose-dependent manner similar to that of gallic acid (50 μg/mL).

Superoxide dismutase (SOD), catalase, glutathione peroxidase (GPx), and glutathione (GSH) are considered as first-line antioxidant defense systems and play an important role in maintaining the cellular redox environment [41]. As demonstrated in Figure 5C,D, *t*-BHP treatment significantly ameliorated the levels of SOD1, catalase, and GPx-1 protein, whereas pretreatment with NNSE significantly reversed this trend in a concentration-dependent manner. The endogenous antioxidant protein levels were also induced by gallic acid in the t-BHP model. These data support the enhancement of antioxidant enzyme proteins by NNSE, resulting in the maintenance of the cellular redox balance and attenuating oxidative stress-induced cell death. Further evidence has revealed that polyphenolic compound-rich medicinal plants/food can ameliorate SOD1, CAT, and GPx activity to minimize oxidative stress [42,43]. Our previous report revealed that a methanolic extract of the *N. nouchali* flower and leaves increased the transcription and translation of the SOD, CAT, and GPx enzymes [4,29]. Furthermore, mounting evidence suggests that the administration of *N. alba, N. pubescens,* and *N. stellata* flowers attenuates hepatotoxicity by augmenting the activity of endogenous enzymes such as SOD, CAT, and GPx [33,44,45].

Superoxide dismutase (SOD), catalase, glutathione peroxidase (GPx), and glutathione (GSH) are considered as first-line antioxidant defense systems and play an important role in maintaining the cellular redox environment [41]. As demonstrated in Figure 5C,D, *t*-BHP treatment significantly ameliorated the levels of SOD1, catalase, and GPx-1 protein, whereas pretreatment with NNSE significantly reversed this trend in a concentration-dependent manner. The endogenous antioxidant protein levels were also induced by gallic acid in the t-BHP model. These data support the enhancement of antioxidant enzyme proteins by NNSE, resulting in the maintenance of the cellular redox balance and attenuating oxidative stress-induced cell death. Further evidence has revealed that polyphenolic compound-rich medicinal plants/food can ameliorate SOD1, CAT, and GPx activity to minimize oxidative stress [42,43]. Our previous report revealed that a methanolic extract of the *N. nouchali* flower and leaves increased the transcription and translation of the SOD, CAT, and GPx enzymes [4,29]. Furthermore, mounting evidence suggests that the administration of *N. alba, N. pubescens,* and *N. stellata* flowers attenuates hepatotoxicity by augmenting the activity of endogenous enzymes such as SOD, CAT, and GPx [33,44,45].

Multiple lines of evidence recommend that polyphenolics, such as protocatechuic acid, vanillic acid, gallic acid, naringenin, 5-*O*-caffeoylquinic acid, catechin, taxifolin, and vicenin-2 have the capability to augment the endogenous antioxidant system, leading to cellular protection from oxidative stress [46,47,48,49,50,51,52]. Therefore, it is hypothesized that the augmentation of first-line antioxidant enzymes by NNSE, resulting from an abundance of phenolics and flavonoids, may contribute to the beneficial effects of NNSE against oxidative stress.

### 3.4. NNSE Induces Phase II Enzymes through Nrf2 Regulation

**Figure 6:**
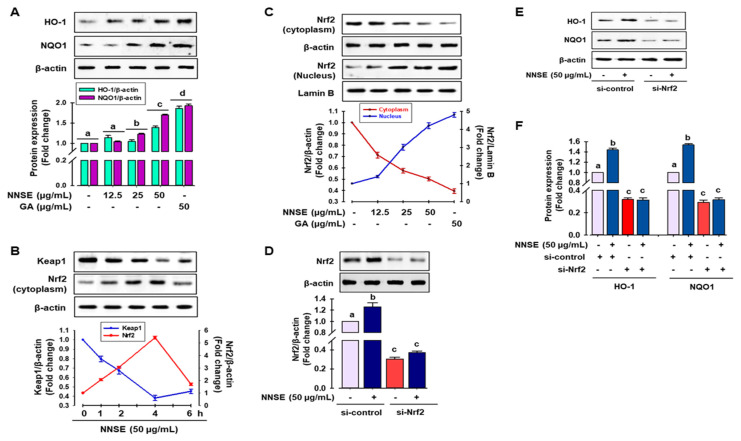
NNSE induces phase II antioxidant enzymes through Nrf2 activation. (**A**) RAW 264.7 cells were treated with NNSE for 24 h, and phase II antioxidant enzyme expression was determined by immunoblotting assay. (**B**) RAW 264.7 cells were treated with NNSE (50 µg/mL) for various times, and the levels of cytoplasmic Keap1 and Nrf2 protein were confirmed by western blot analysis. (**C**) Cells were treated with NNSE and gallic acid for 4 h, and nuclear translocation of Nrf2 was measured by immunoblotting assay. Cells were treated with NNSE in the presence or absence of si-Nrf2 RNA, and the level of (**D**) Nrf2, and (**E**) HO-1, and NQO1 protein was measured by western blot analysis. (**F**) The relative expression of HO-1 and NQO1 protein was quantified by Image J software. Values are expressed as the mean ± SD (*n* = 3), and different letters are considered statistically significant (*p* < 0.05) to one another. GA, gallic acid.

Several reports have indicated that phase II detoxifying/antioxidant enzymes, such as heme oxygenase-1 (HO-1) and nicotinamide adenine dinucleotide (phosphate) (NAD(P)H) quinone oxidoreductase-1 (NQO1), play an important role in detoxifying ROS and is modulated by Nrf2, a central regulator of ARE-driven antioxidant gene expression [53]. Accordingly, to judge whether NNSE has the potential to boost phase II antioxidant enzymes through the regulation of Nrf2, immunoblotting analysis was performed. As shown in Figure 6A, NNSE treatment significantly increased the levels of HO-1 and NQO1 protein in a dose-dependent manner, similar to that of gallic acid. In a resting state, Nrf2 activity is firmly controlled in the cytosol by Kelch-like ECH associating protein 1 (Keap1) as an adaptor protein for Cullin-3 (Cul3)-dependent E3 ubiquitin ligase enzyme, which is responsible for Nrf2 ubiquitination and degradation [4,53]. Thus, western blot analysis was performed to evaluate the function of NNSE in preventing cytosolic Nrf2 degradation and enhancing the nuclear translocation of Nrf2. Figure 6B indicates the time-dependent attenuation of Keap1 protein in the cytoplasm in association with Nrf2 enrichment by NNSE treatment, which peaked at 4 h. In addition, NNSE treatment significantly increased nuclear Nrf2 content in association with minimized cyto-Nrf2 levels (Figure 6C). Furthermore, to validate the potential of NNSE to stimulate phase II antioxidant enzymes by modulating Nrf2, knocked-down expression of Nrf2 using a small interfering RNA (siRNA) technique was performed (Supplementary Figure S3). Nrf2 protein levels were considerably reduced by si-Nrf2 treatment, which was not restored even after treatment with NNSE (Figure 6D). Furthermore, as shown in Figure 6E,F, si-Nrf2 treatment significantly reduced the levels of HO-1 and NQO1 proteins, and NNSE treatment was unable to normalize basal HO-1 and NQO1 protein levels. This observation indicates that NNSE may disrupt the proteasomal degradation of Nrf2 in the cytoplasm by Keap1 and may facilitate the nuclear translocation of Nrf2, resulting in upregulation of HO-1 expression.

Several studies have revealed that extracts from various medicinal plants/food, such as *N. nouchali* flower and leaves, *N. alba, N. lotus, N. pubescens,* and *N. stellata* flower extract, result in the activation of Nrf2-mediated phase II enzyme expression in Raw 264.7 cells [4,29,33]. Furthermore, protocatechuic acid, vanillic acid, gallic acid, naringenin, 5-*O*-caffeoylquinic acid, catechin, taxifolin, and vicenin-2 can modulate the Nrf2/ARE/HO-1 signaling cascade and attenuate oxidative stress-mediated kidney and hepatic cell death [46,47,48,52].

### 3.5. NNSE Activates MAPKs and Regulates Nuclear Translocation of Nrf2, Leading to Reduced Oxidative Stress

**Figure 7:**
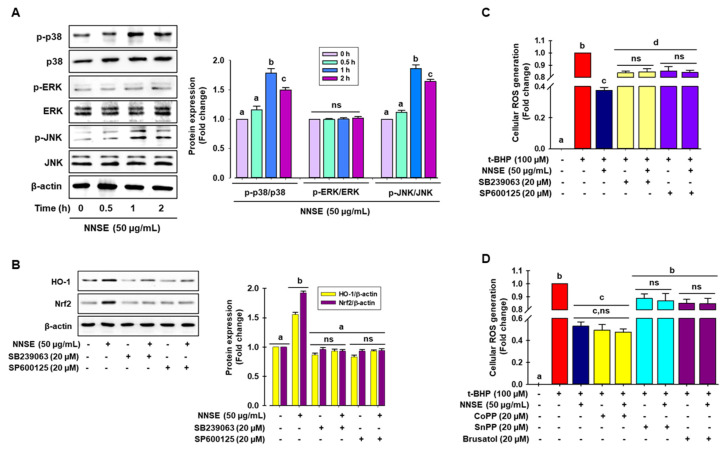
Activation of p38 and JNK by NNSE results in Nrf2 translocation. (**A**) RAW 264.7 cells were treated with NNSE (50 µg/mL) for various times, and kinase activity was determined by immunoblot assay. (**B**) Cells were treated with NNSE and specific inhibitors, SB239063 (p38 inhibitor) and SP600125 (JNK inhibitor), for 1 h, and Nrf2 and HO-1 protein levels were analyzed by western blot analysis. (**C**) Cells were treated with NNSE and the specific inhibitors, SB239063 and SP600125, and cellular ROS generation was quantified by the DCFH-DA methods. (**D**) Cells were treated with NNSE and CoPP (HO-1 activator), SnPP (HO-1 inhibitor), and brusatol (Nrf2 inhibitor), and cellular ROS generation was quantified by the DCFH-DA methods. Values are expressed as the mean ± SD (*n* = 3), and different letters are considered statistically significant (*p* < 0.05) to one another.

Many studies have demonstrated that phosphorylation of MAPKs, such as ERK, JNK, and p38, can positively regulate phase II antioxidant enzyme expression by activating the ARE/Nrf2 mechanism in various cell types [54,55]. Here, immunoblot analysis was performed to identify the signaling pathways participating in the regulation of phase II antioxidant enzyme activity in NNSE-treated cells. As presented in Figure 7A, NNSE treatment significantly enhanced the phosphorylation of p38 and JNK from 0.5 to 2 h, with a peak at 1 h, whereas NNSE treatment did not result in ERK phosphorylation. To determine whether p38 and JNK can regulate HO-1 expression by modulating Nrf2, cells were treated with each specific inhibitor before stimulation with NNSE. As shown in Figure 7B, both the p38 and JNK inhibitor (SB239063 and SP600125, respectively) markedly suppressed HO-1 and Nrf2 expression, which was increased by NNSE treatment. This indicates that p38 and JNK phosphorylation may regulate the induction of HO-1 through modulation of Nrf2 signaling in RAW 264.7 cells. Furthermore, to validate the role of MAPK/Nrf2/HO-1 signaling in the reduction in oxidative stress, cells were treated with each specific inhibitor before stimulation with NNSE, and intracellular ROS generation induced by t-BHP treatment was measured. Interestingly, t-BHP stimulation significantly increased cellular ROS generation, which was strongly ameliorated by NNSE (Figure 7C,D, third column), whereas treatment with p38 and JNK inhibitors (SB239063 and SP600125, respectively) reversed this trend (Figure 7C). Moreover, treatment with the HO-1 inducer, CoPP, strongly and significantly abolished t-BHP-induced generation of cellular ROS, and this trend was reversed by HO-1 and Nrf2 inhibitors (SnPP and Brusatol, respectively) (Figure 7D). These data strongly suggest that phosphorylation of p38 and JNK by NNSE can regulate Nrf2/HO-1 signaling, which accounts for cell subsistence against oxidative stress in RAW 264.7 cells.

## 4. Conclusions

Oxidative stress is considered one of the major contributing factors to the development and progression of several acute and chronic disorders. Thus, it is anticipated that antioxidants may have valuable health effects as prophylactic agents. Here, ESI-MS/MS analysis revealed the abundance of secondary metabolites in NNSE and demonstrated excellent antioxidant activity in cell-free assays and at the cellular level. Furthermore, NNSE has the potential to reduce the oxidative burden by attenuating the first-line antioxidant system and activating the MAPK-Nrf2-HO-1 signaling cascade, resulting in the suppression of cellular ROS generation. Our findings provide new insights into the cytoprotective effects and mechanisms of *Nymphaea nouchali* stem extract against oxidative stress, which may be a useful remedy for oxidative stress-induced disorders.

## Figures and Tables

**Table 1:** Secondary metabolites identified in *Nympheae nouchali* stem using negative-mode electrospray ionization mass spectrometry (ESI-MS/MS).

Groups	No.	Compound Name	EF	Observed *m/z*	Calculated *m/z*	Adducts	MS/MS Fragments	CE (eV)
Phenolic acids	1	Salicylic acid	C_7_H_6_O_3_	137.0227	137.0238	[M − H]^−^	93.03	10
	2	Methyl benzoic acid	C_8_H_8_O_2_	135.0444	135.0446		91.05	10
	3	Protocatechuic acid	C_7_H_6_O_4_	153.0186	153.0187		135.00,109.02	10
	4	Vanillic acid	C_8_H_8_O_4_	167.0344	167.0344		151.00, 123.04,107.01	10
	5	Gallic acid	C_7_H_6_O_5_	169.0134	169.0137		125.02	10
	6	Methoxygallate	C_8_H_8_O_5_	183.0290	183.0293		166.99, 139.04, 123.01, 111.01	30
	7	Brevifolincarboxylic acid	C_13_H_8_O_8_	291.0141	291.0140		247.02,219.02,203.03,191.03,175.04	20
	8	Pyrogallol gallate	C_13_H_10_O_8_	293.0300	293.0297		169.01, 125.02	10
	9	*p*-coumaroyltartaric acid	C_13_H_12_O_8_	295.0456	295.0453		163.04,149.00,145.02	20
	10	Methyl brevifolincarboxylic acid	C_14_H_10_O_8_	305.0300	305.0297		245.00,217.01, 201.01, 189.01,161.02,145.02	20
	11	Galloylglucose	C_13_H_16_O_10_	331.0667	331.0665		241.03, 211.02, 169.01, 125.02	20
	12	Niazinin B	C_15_H_21_NO_6_S	342.1115	342.1011		310.07,196.04,166.03,164.01	20
	13	Lalioside	C_14_H_18_O_10_	345.0822	345.0822		301.05,211.02,183.02,165.01	20
	14	5-O-caffeoylquinic acid	C_16_H_18_O_9_	353.0865	353.0872		309.09,191.05,179.03,173.04,161.02	20
	15	3-Feruloylquinic acid	C_17_H_20_O_9_	367.1050	367.1029		193.05, 173.04	20
	16	Isoferulic acid 3-O-glucuronide	C_16_H_18_O_10_	369.0822	369.0821		193.05,177.04	10
	17	Ethyl 5-O-caffeoylquinic acid	C_18_H_22_O_9_	381.1204	381.1186		201.07,191.05,179.03,161.02	20
	18	Gallic acid-O-rutinoside	C_19_H_26_O_14_	477.1249	477.1244		315.07, 297.06, 283.04, 211.02, 169.01, 105.05, 125.02, 93.03	30
	19	Digalloylglucose	C_20_H_20_O_14_	483.0779	483.0774		331.06, 313.05, 303.07, 271.04, 241.03, 211.02, 169.01, 125.02	30
Flavonoids	20	Catechin	C_15_H_14_O_6_	289.0692	289.0712	[M − H]^−^	245.04, 205.05,151.04,137.02	30
	21	Taxifolin	C_15_H_12_O_7_	303.0470	303.0504		285.04,241.05,177.01,151.04	20
	22	Elephantorrhizol	C_15_H_14_O_8_	321.0628	321.0616		303.05,169.01,151.04	30
	23	Naringenin-7-sulfate	C_15_H_12_O_8_S	351.0175	351.0174		271	30
	24	Apigenin-6-C-galactoside-8-C-arabinoside	C_26_H_28_O_14_	563.1405	563.1400		545.13, 503.11, 473.10, 443.09, 383.07, 353.06	40
	25	Apigenin 6,8-di-C-glucoside (Vicenin-2)	C_27_H_30_O_15_	593.1517	593.1506		503.11,473.10, 383.07,353.06	40
	26	Quercetin-3-neohesperidoside	C_27_H_30_O_16_	609.1460	609.1455		245.04, 205.05,151.04,137.02	30
Sugars	27	L-arabinofuranose	C_5_H_10_O_5_	149.0445	149.0450	[M − H]^−^	131.03, 89.02, 75.01, 71.01	10
	28	Glucose	C_6_H_12_O_6_	179.0553	179.0555	[M − H]^−^	113.02, 101.02, 89.02, 71.01	10
				215.0322	215.0324	[M + Cl]^−^	179.05, 113.02, 101.02, 89.02, 71.01	10
				217.0293	217.0295	[M + K-2H]−		
	29	6-O-β-D-galactopyranosyl-D-galactose	C_12_H_22_O_11_	341.1087	341.1083	[M − H]^−^	179.05, 113.02, 101.02, 89.02, 71.01	10
				377.0854	377.0857	[M + Cl]^−^	341.10, 179.05, 113.02, 101.02, 89.02, 71.01	20
				379.2159	379.2161	[M + K-2H]^−^		
Dicarboxylic acids	30	Succinic acid	C_4_H_6_O_4_	117.0175	117.0187	[M − H]^−^	99.00, 73.02	10
	31	Malic Acid	C_4_H_6_O_5_	133.0124	133.0137		115.00, 89.02, 71.01	10
	32	Citramalic acid	C_5_H_8_O_5_	147.0292	147.0293		133.01, 115.00, 87.00	20
	33	Hydroxyadipic acid	C_6_H_10_O_5_	161.0450	161.0450		143.03, 101.02, 99.04	20
Amino acids	34	α-amino-β-hydroxybutyric acid	C_4_H_9_NO_3_	118.0141	118.0140	[M − H]^−^	100.04, 96.00, 74.02	10
	35	Pyroglutamic acid	C_5_H_7_NO_3_	128.0336	128.0347		82.03, 71.01, 69.00	20
Flavoring agents	36	Maltol	C_6_H_6_O_3_	125.0227	125.0238	[M − H]^−^	97.03, 95.01, 83.04, 79.01,	20
	37	Kahweofuran	C_7_H_8_OS	139.0213	139.0217		111.02, 109.01, 68.98, 67.01	20
	38	Cetone V	C_16_H_24_O	231.1748	231.1748		173.09, 155.08, 137.13,93.03	20
Fatty acids	39	Methylcaproic acid	C_7_H_14_O_2_	129.0916	129.0915	[M − H]^−^	99.04, 85.10, 71.01, 69.07	20
	40	Ethyl-β-hydroxybutyric acid	C_6_H_12_O_3_	131.0696	131.0708		113.06, 87.08, 85.06	10
	41	Caprylic acid	C_8_H_16_O_2_	143.1061	143.1072		125.10, 113.06, 99.05, 85.03,	10
	42	Dodecadienoic acid	C_12_H_20_O_2_	195.1384	195.1385		179.11, 161.10, 97.10, 71.09	30
	43	Dodecanoic acid	C_12_H_24_O_2_	199.1697	199.1698		181.16, 165.13, 163.11, 139.11, 135.11	20
Sialic acid	44	2-Deoxy-2,3-dehydro-N-acetylneuraminic acid	C_11_H_17_NO_8_	290.0877	290.0875	[M − H]^−^	200.05, 170.04, 128.07	10
Terpenoid	45	α-γ-Onoceradienedione	C_30_H_46_O_2_	437.3421	437.3419	[M − H]^−^	219.17, 205.15, 95.08	40
others	46	Glyceric acid	C_3_H_6_O_4_	105.0184	105.0187	[M − H]^−^	87.00, 75.00, 61.03	10
	47	Sorbic acid	C_6_H_8_O_2_	111.0443	111.0446		67.05	10
	48	Salicylaldehyde	C_7_H_6_O_2_	121.0278	121.0289		93.03, 65.03	30
	49	Methyl benzoic acid	C_8_H_8_O_2_	135.0444	135.0446		91.05	10
	50	Hydroxynicotinic acid	C_6_H_5_NO_3_	138.0189	138.0191		94.02	20
	51	Ribonic acid	C_5_H_10_O_6_	165.0395	165.0399		149.04, 105.01, 87.00, 75.00	10
	52	Shikimic acid	C_7_H_10_O_5_	173.0445	173.0450		155.03, 137.02, 111.04, 93.03	10
	53	Quinic acid	C_7_H_12_O_6_	191.0553	191.0555		173.05, 127.04, 93.03, 85.03	10
	54	N-undecanoylglycine	C_13_H_25_NO_3_	242.1756	242.1756		224.1656, 182.1550	20

EF, elemental formula, CE, collision energy.

## Data Availability

Data is contained within the article.

## Supplementary Materials

The following are available online at https://www.mdpi.com/article/10.3390/antiox10050719/s1, Figure S1: Total phenolic and flavonoid content of NNSE, Figure S2: Effect of NNSE on cell viability in RAW264.7 cells, Figure S3: Nrf2 expression using si-RNA in RAW264.7 cells, Table S1. List of the primary antibodies used in the study.
