# Supplementary material for: High Resolution Mass Spectroscopy-Based Secondary Metabolite Profiling of Nymphaea nouchali (Burm. f) Stem Attenuates Oxidative Stress via Regulation of MAPK/Nrf2/HO-1/ROS Pathway
Source: Antioxidants (Basel). 2021 May 3;10(5):719. doi: 10.3390/antiox10050719 (PMC8147620; doi:10.3390/antiox10050719)
Supplement: Supplementary file 1 [file antioxidants-10-00719-s001.zip › antioxidants-1189745-supplementary.pdf]

Supplementary Data

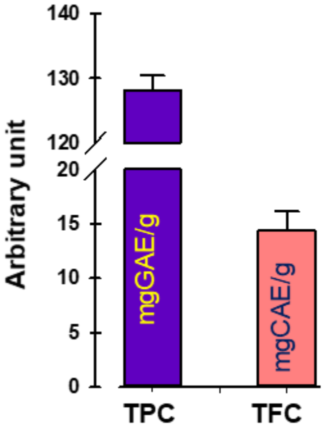

Figure S1: Total phenolic and flavonoid content of NNSE
TPC: Total phenolic content (mgGAE/g extract)
TFC: Total flavonoid content (mgCAE/g extract)

Figure S1

Supplementary Data

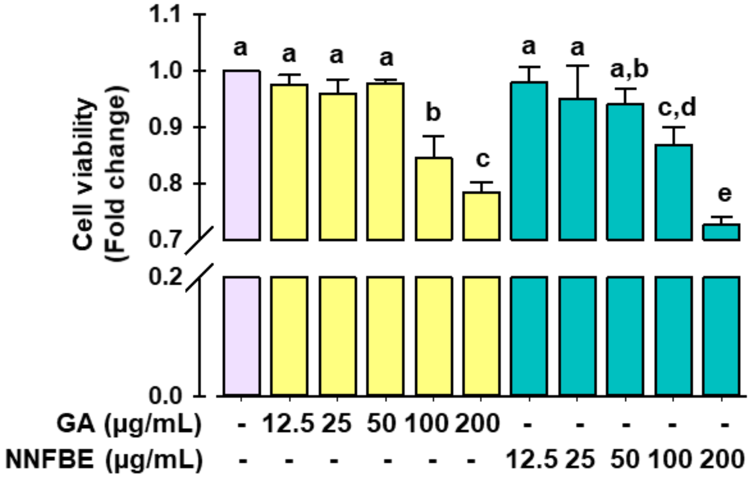

Figure S2: Effect of NNSE on cell viability in RAW264.7 cells. Values are expressed as the mean ± SD (n = 3) and different letters are considered as statistically significant ($p < 0.05$) to each other. GA: gallic acid;

Figure S2

Supplementary Data

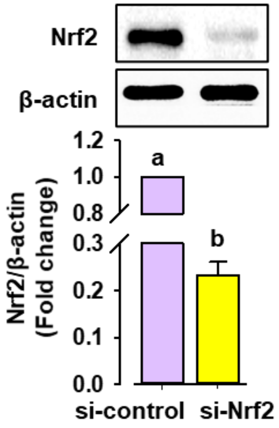

Figure S3: Nrf2 expression using si-RNA in RAW264.7 cells. Values are expressed as the mean ± SD (n = 3) and different letters are considered as statistically significant ($p < 0.05$) to each other.

Figure S3

Table S1: List of the primary antibodies used in the study.

Name	Catalog no.	Company	Antigen	Host	Dilutions	Membrane
Anti-SOD1	BS91268	Bioworld Technology, Inc.	SOD1	Rabbit	1:1000	Nitrocellulose membrane
Anti-catalase	BS90194	Bioworld Technology, Inc.	Catalase	Rabbit	1:1000	Nitrocellulose membrane
anti-GPx-1	MB9027	Bioworld Technology, Inc.	GPx-1	Mouse	1:1000	Nitrocellulose membrane
Anti-HO-1	sc-136256	Santa Cruz Biotechnology, Inc.	HO-1	Mouse	1:1000	Nitrocellulose membrane
Anti Nrf2	sc-81342	Santa Cruz Biotechnology, Inc.	Nrf2	Mouse	1:1000	Nitrocellulose membrane
Anti-Lamin B	BS3547	Bioworld Technology, Inc.	Lamin B	Rabbit	1:1000	Nitrocellulose membrane
Anti-p-p38	sc-166182	Santa Cruz Biotechnology, Inc.	p38	Mouse	1:1000	Nitrocellulose membrane
Anti-p38	BS3567	Bioworld Technology, Inc.	p38	Rabbit	1:1000	Nitrocellulose membrane
Anti-p-ERK1/2	sc-7383	Santa Cruz Biotechnology, Inc.	ERK	Mouse	1:1000	Nitrocellulose membrane
Anti-ERK1/2	BS 6472	Bioworld Technology, Inc.	ERK	Rabbit	1:1000	Nitrocellulose membrane
Anti-p-JNK	BS 4322	Bioworld Technology, Inc.	JNK	Rabbit	1:1000	Nitrocellulose membrane
Anti-JNK	sc-7345	Santa Cruz Biotechnology, Inc.	JNK	Mouse	1:1000	Nitrocellulose membrane
Anti- βactin	Sc-47778	Santa Cruz Biotechnology, Inc.	β-actin	Mouse	1:1000	Nitrocellulose membrane
